# Human-modified biogeographic patterns and conservation in game birds: The dilemma of the black francolin (*Francolinus francolinus*, Phasianidae) in Pakistan

**DOI:** 10.1371/journal.pone.0205059

**Published:** 2018-10-05

**Authors:** Giovanni Forcina, Monica Guerrini, Imran Khaliq, Aleem Ahmed Khan, Filippo Barbanera

**Affiliations:** 1 Department of Biology, University of Pisa, Pisa, Italy; 2 Department of Zoology, Ghazi University, Dera Ghazi Khan, Punjab, Pakistan; University of Innsbruck, AUSTRIA

## Abstract

The ever-increasing human-mediated wildlife reshuffling is raising concern for the conservation of biodiversity. The loss of biological distinctiveness among regions lessens the genetic diversity and consequently the evolutionary potential of local biotas to tackle present-day global change and human disturbance. This process may be sometimes cryptic unless investigated by means of a molecular approach. In this respect, game birds are a paradigmatic case. The black francolin (*Francolinus francolinus*, Phasianidae) is a medium-sized galliform whose distribution range stretches from Cyprus to the Gulf of Bengal. Six morphologic subspecies are known, with three of which occurring in Pakistan, where the species is heavily hunted and used as pet for chirping competitions. We genotyped 98 samples (feathers) at both the entire mitochondrial DNA Control Region gene and nine microsatellite loci to get a deeper insight into the genetic diversity of the black francolin in Pakistan in order to offer cogent recommendations for its conservation management. We identified several mtDNA lineages that were consistent with the currently described subspecies/taxonomy whose pattern of co-occurrence is compatible with the geological history and the faunal movement routes of the region under study. However, the biparentally inherited microsatellites returned a quite discordant picture of an extensive, sex-biased genetic mixing due to the intensive relocations of already overharvested male individuals for chirping competitions. Our results indicated that the genetic integrity of the black francolin in Pakistan could be seriously at risk and call for monitoring and limiting its trade other than enhancing the public awareness of the importance of local biodiversity resources.

## Introduction

Since the Bronze Age, human activities have been shaping the distribution of a variety of taxa with an increasing movement of faunal and floral assemblages worldwide [1]. In the last decades, natural and artificial breeding have enabled massive wildlife relocations either for harvesting purposes (hunting, fishing) or for sustaining populations of non-game species. This impressive rate of wildlife relocation is raising increasing conservation concern, as the consequential reshuffling may affect taxa in the wild to such an extent that it results into the “loss of biological distinctiveness among regions following the replacement of native biotas by locally expanding non-natives” [2]. This phenomenon can eventually impair the delivery of supporting ecosystem services, of which genetic diversity is sometimes referred to as the most fundamental [3]. In fact, the release of individuals into non-native grounds may cause adverse changes such as loss of genetic variation and behavioural traits, alteration of population structure, and hybridisation with domesticated relatives, conspecifics of non-local origin or exotics [4–8]. Even though genetic mixing may prove beneficial under some circumstances (e.g., genetic rescue) [9], it often results into outbreeding depression [10]. In this respect, game birds are a paradigmatic case [11] and Phasianidae one of the most problematic groups [12–16].

The black francolin (*Francolinus francolinus*, Phasianidae) is a medium-sized galliform whose distribution range stretches from Cyprus to the Gulf of Bengal [17–19]. Among the six recognized morphological subspecies, two are traditionally known to occur in Pakistan: *F*. *f*. *bogdanovi*, from the South West to the North East, and the endemic *F*. *f*. *henrici* mostly in the North of the country [20]. The black francolin is one of the most valued game birds of Pakistan and a strong hunting pressure drove the species towards a sharp drop in numbers across the country [21–23]. Recent estimates indicated average density ranges between 0.6 and 4.4 birds/Km^2^ in the different habitats of Mang Game Reserve in Haripur (North Pakistan) [24], peaking up to around 6 and 8 birds/Km^2^ in Lehri Nature Park (North Pakistan) [25] and Lal Suhanra National Park (Central Pakistan) [26], respectively. Even though pre-decline estimates for these regions are missing, numbers are pretty low when compared with the 14–33 birds/Km^2^ recorded for the healthy insular population inhabiting Cyprus [27].

The black francolin has been held in high regard for its delicate flavour since the Classic Age and is presently an important source of bush meat for poor people in Asia [28, 29]. Poaching represents a serious threat to the survival of the black francolin together with habitat transformation and the use of pesticide/herbicidal sprays in agricultural practices [22, 30]. Nevertheless, in Pakistan the species, also known as black partridge, is largely welcomed by farmers as it feeds on insect pests [31], and by the broader villagers as pets for chirping competitions (“All Pakistan Black Partridge Chirping Competitions”) among male birds [23]. Also, the meat of the black francolin is locally praised as a powerful aphrodisiac [32].

Genetic information is fundamental to plan management strategies for the conservation of a given species. For the black francolin of Pakistan, first data came to the light only recently, with Riaz et al. [23] suggesting a high level of diversity. However, this study relied on a small sample size (*N* = 23) with records collected exclusively in the central provinces of Punjab and Balochistan. Moreover, the authors used Random Amplified Polymorphic DNA (RAPD) markers, whose suitability in the investigation of genetic variability has been called into question since Pérez et al. [33]. Later on, Forcina et al. [34] proved that a third subspecies, the *F*. *f*. *asiae*, occurs in both North East and South East Pakistan. Despite the relatively large number of samples (*N* = 75) collected across the entire country, the black francolin was investigated at the mitochondrial DNA only. Uncertainty thus remains regarding the spatial genetic structure of the species.

We assumed that in the absence of physical barriers, genetic admixture among the three subspecies is likely and no overt genetic structure is expected. To test our hypothesis, we characterized the genetic diversity of the black francolin in Pakistan in order to gain data useful for implementing its sustainable use and long-term protection within an evolutionary adaptive framework [35]. We used molecular sexing and genotyped each bird at both the mitochondrial (mtDNA) and nuclear microsatellite (Short Tandem Repeats, STR) DNA, the complementary nature of these markers being deemed essential to address our goal [36].

## Materials and methods

### Biological sampling and animal ethics

**Fig 1:**
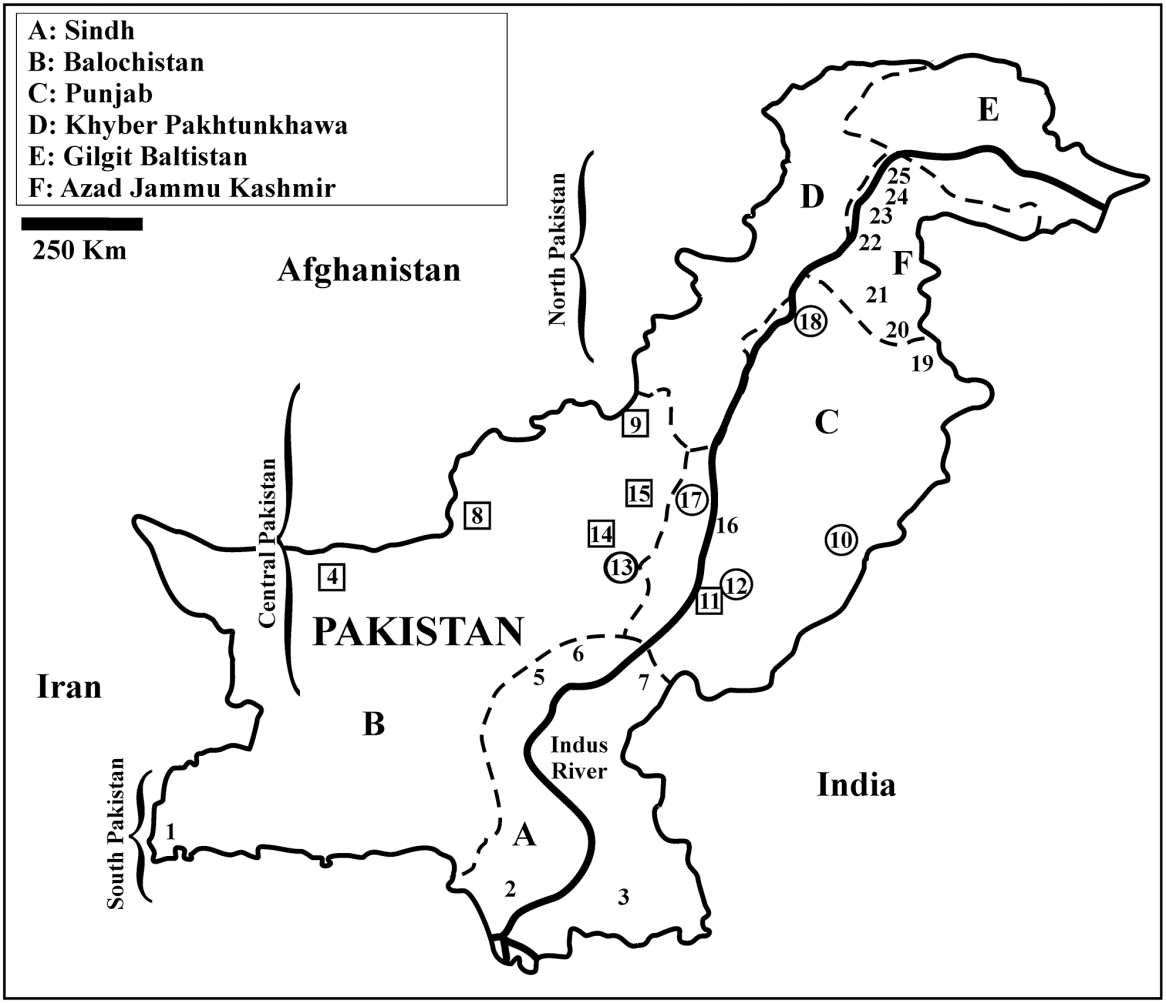
Map of the study area with sampling localities (1 to 25) across Pakistani provinces (see inset). Open circles mark out the populations investigated also in the paper of Riaz et al. [23]; open squares indicate the six populations that are new to the present study when compared to the previous one from the same group of authors [34]. See also S1 Table for details on the sample size.

Between 2008 and 2014, 98 samples (feathers) of wild black francolins were collected in Pakistan (North: *N* = 21; Central: *N* = 55; South: *N* = 22) from 25 populations ranging from the Indian Ocean across the Indus River valleys to the foothills of the Himalayas. Of these, 75 were already investigated only at the mtDNA by Forcina et al. [34] while the remaining 23 are new to this study and include six populations not investigated earlier (Basti, *N* = 6; Musa Khel, *N* = 3; Mekhtar, *N* = 2; Quetta, *N* = 3; Zhoab, *N* = 2 and Chagai, *N* = 3: Fig 1). The large majority of samples was obtained from trapped birds. Both protocol and procedures employed were ethically reviewed and approved by the Departmental Ethical Research Committee of the Department of Zoology of Ghazi University (#GUDGK/Zool./67, Dera Ghazi Khan, Pakistan), and they were in accordance to the animal health and welfare rules under the Punjab Wildlife Conservation Act (1974). Black francolins were hunted only in the area of Badin (Sindh), with one sample kept from each trip to lessen the risk of genotyping individuals from the same covey (Fig 1; S1 Table in the Supporting Information).

### DNA extraction

DNA was extracted from feathers using the Puregene Core Kit-A (Qiagen, Hilden Germany) and following the manufacturer’s instructions. A 3 mm-long fragment from the base of the quill was employed as in Barbanera et al. [37]. The reliability of each DNA extraction was monitored through two negative controls (no tissue added). The DNA content and purity was determined with an Eppendorf BioPhotometer (AG Eppendorf, Hamburg, Germany).

### PCR-based sex determination

The sex of each francolin was inferred using a PCR-based method. Chromo Helicase DNA (CHD) gene of ZZ (males) and ZW (females) sexual chromosomes was amplified with primers USP1, USP3, CPE15F and CPE15R [38, 39] previously isolated from chicken (*Gallus gallus*) genome. Amplifications (25 μl) were performed in a T100 thermal cycler (BioRad, Hercules, California, USA) with 1 μl of *Taq* DNA Polymerase (1 U/μl, Sigma Aldrich, Saint Louis, Missouri, USA), 2.5 μl of 2.5 mM dNTP, 2.5 μL of 10X PCR buffer, 2 μl of 25 mM MgCl_2_, 1.5 μl of each primer (1 μM) and 10 ng of DNA. PCR thermal profile was as follows: 3 min at 94°C, then 35 cycles of 1 min and 20 s at 94°C, 1 min and 30 s at 60°C and 1 min at 72°C, followed by 7 min at 72°C. When PCR products were visualized by means of a gel electrophoresis, all individuals showing one or two bands were identified as males (homogametic sex) and female (heterogametic sex), respectively. The shorter band (250 bp, W chromosome) was shared between sexes as opposed to the larger one (370 bp, Z chromosome).

### Mitochondrial DNA amplification and sequencing

The entire mtDNA Control Region (CR) gene (ca. 1170 bp) was amplified using primers PHDL (forward) of Fumihito et al. [40] and H1321 (reverse) of Barbanera et al. [37]. Reactions (50 μl) were prepared using 4 μl of 25 mM MgCl_2_, 5 μl of 10X PCR Gold buffer, 5 μl of 2.5 mM dNTP and 1 μl of Ampli*Taq* Gold DNA Polymerase (1 U/μl, Thermo Fisher Scientific, Waltham, Massachusetts, USA), 3 μl of each primer (1 μM) and 20 ng of DNA template. Thermal profile was as follows: 3 min at 94°C, then 30 cycles of 1 min at 94°C, 2 min at 55 °C and 1 min at 72°C followed by 7 min at 72°C. In case of very old feathers, we obtained the entire CR gene using the purified products (Genelute PCR Clean-up Kit) of the first amplification as DNA template in two semi-nested PCRs (snPCRs) [41]. In the snPCRs, two overlapping fragments (1^st^: 628 bp; 2^nd^: 699 bp) were amplified for each sample in two reaction tubes by applying the same thermal profile as in the first PCR. Final PCR products were purified as above and sequenced on both DNA strands (BigDye Terminator v. 3.1 Cycle Sequencing Kit, ABI 3730 DNA automated sequencer, Thermo Fisher Scientific) at Genechron (ENEA, Rome).

### Mitochondrial DNA analysis

A first mtDNA alignment (entire CR gene, ca. 1170 bp) included 98 sequences from birds sampled in Pakistan and those retrieved from 124 samples (total, 98 + 124 = 222) collected across the whole distribution range of *F*. *francolinus* and previously analysed by Forcina et al. [34] (S1 Table). Relying on the largest and most informative modern black francolin DNA dataset available to date, this alignment allowed us to assign Pakistani individuals to a given subspecies. A second mtDNA alignment (partial CR gene, 185 bp: positions 151–335) was created adding 76 sequences obtained from archival samples of black francolins resident in European and US museum collections [29] to the previous 222 ones (total, 76 + 222 = 298). In particular, this modern + archival partial mtDNA alignment included birds from Pakistan (*N* = 3), India (*N* = 20), Nepal (*N* = 7), and Bangladesh (*N* = 1), thus bearing potential for shedding further light on the biogeography of the black francolin across the entire Indian sub-continent. Despite its shortness, this CR fragment had proven to hold a still fairly high resolution power [29].

We performed both alignments with clustalx 1.81 [42] and used arlequin 3.5.1 [43] to infer haplotypes (H and h, for entire and partial mtDNA CR gene alignment, respectively). Then, additional molecular computations were performed only for the first alignment. We built a network using the Median Joining method [44] implemented in network 5.0.0.3 (2004–2018 Fluxus Technology). Only for the population of Pakistan, we used arlequin to calculate haplotype diversity (*h*), nucleotide diversity (π), number of pairwise differences (*k*), and to investigate the partition of the mtDNA diversity (Analysis of the Molecular Variance, amova) among and within regions (North, Central and South Pakistan) using the *ϕ*_st_ analogous of Wright’s *F*-statistics [45].

### Microsatellite DNA

All 98 Pakistani samples were genotyped at nine STR loci using primers isolated from either chicken or red-legged partridge (*Alectoris rufa*) genome and previously applied in the black francolin by Forcina et al. [46], who provided both PCR set-up and thermal profiles in detail. We also genotyped at the same STR panel a few black francolins from Iran (*N* = 4: haplotypes H20, H21 and H23), Afghanistan (*N* = 1: H21), India (*N* = 3: H5 and H6) and Nepal (*N* = 14: H2, H3 and H4). We used these birds as genetic reference for *F*. *f*. *bogdanovi* (Iran, Afghanistan) and *F*. *f*. *asiae* (India, Nepal) in the microsatellite DNA data computations. All cross-amplifications were carried out as in Barbanera et al. [14], and the gene sizing performed at the Laboratory of Biochemistry of the Department of Pathology (University of Pisa) on an ABI Prism 3730 DNA automated sequencer using genescan (Thermo Fisher).

The STR panel was investigated using micro-checker 2.2.3 [47] to test for null alleles, allele dropout and scoring errors due to stuttering, and the discriminatory power was evaluated with gimlet 1.3.3 [48] (in terms of probability of identity, *P*_ID_, and probability of identity among siblings, *P*_ID_sib) [49]. Analyses were performed using either the whole sample size or males and females separately for North, Central and South Pakistan. We used Arlequin 3.5.1, to: compute the number of alleles per locus, the number of unique alleles and the allelic richness. fstat 2.9.3 [50] was used to compute the Nei’s Index (*I*_n_ or average gene diversity) and the inbreeding coefficient (*f*). We also used genepop 3.4 [51] to calculate expected (*H*_E_) and observed (*H*_O_) heterozygosity and infer deviations from both Hardy-Weinberg Equilibrium (HWE) (global test) and Linkage Disequilibrium (LD) (10,000 dememorizations, 100 batches, 5,000 iterations per batch). The Bonferroni correction [52] was adopted to adjust the significance level of HWE and LD tests. Finally, we used arlequin to investigate the partition of STR diversity using amova and Wright’s *F*-statistics (10000 permutations) [45] and to estimate gene flow (*N*_e_m, effective number of migrants per generation via the private allele method of Slatkin, [53]) among populations inhabiting the same or other regions (e.g., North, Central and South Pakistan: Fig 1).

The genetic diversity of Pakistani populations was also inferred by means of a Bayesian clustering approach using structure 2.3.4 [54]. The number of genetically distinct subpopulations (*K*) was assumed to range from 1 to 10. All simulations were run with 10^6^ Markov Chain Monte Carlo iterations, after a burn-in period of 10^5^ iterations, and replicated 10 times per each *K*-value to check for the consistency of likelihood values between runs. To prevent a biased estimation of *K*, genetic admixture and correlated allele frequencies among subpopulations were included as parameters. The optimal *K* was selected as described by Evanno et al. [55] using structure harvester 0.6.92 [56]. The program clumpp 1.1.2 [57] was used to align the 10 repetitions of the best *K* and get the estimated membership to the *K* clusters for both populations and groups of populations. The assignment threshold (Q_i_) to a given cluster was set to 0.80 [58] and individuals with admixed genotype were unassigned.

Finally, another Bayesian clustering analysis was done including only black francolins from Haroon Abad, Alipur, Rakhni, Bait Suvai, and Chakwal (Fig 1, S1 Table) for purpose of comparison with the results obtained by Riaz et al. [23] who investigated the same populations using RAPDs.

## Results

### PCR-based sex determination

The CHD gene was amplified in all black francolin: 65 birds were identified as males (single PCR product) while 33 as females (two PCR products), with a male/female sex *ratio* of 1.97 (S1 Table).

### Mitochondrial DNA analysis

The entire CR gene alignment included 66 haplotypes (H1-H66: S2 Table), with 63 variable sites (indels comprised). Sixty-one haplotypes were the same as in Forcina et al. [34], while the remaining ones (H14, H33-H35 and H38) were new and held by francolins inhabiting Central Pakistan. The 89.05% of the total mtDNA variability was partitioned within North, Central and South Pakistan and 10.95% across such regions (amova: *ϕ*_st_ = 0.11, *P* < 0.001).

**Fig 2:**
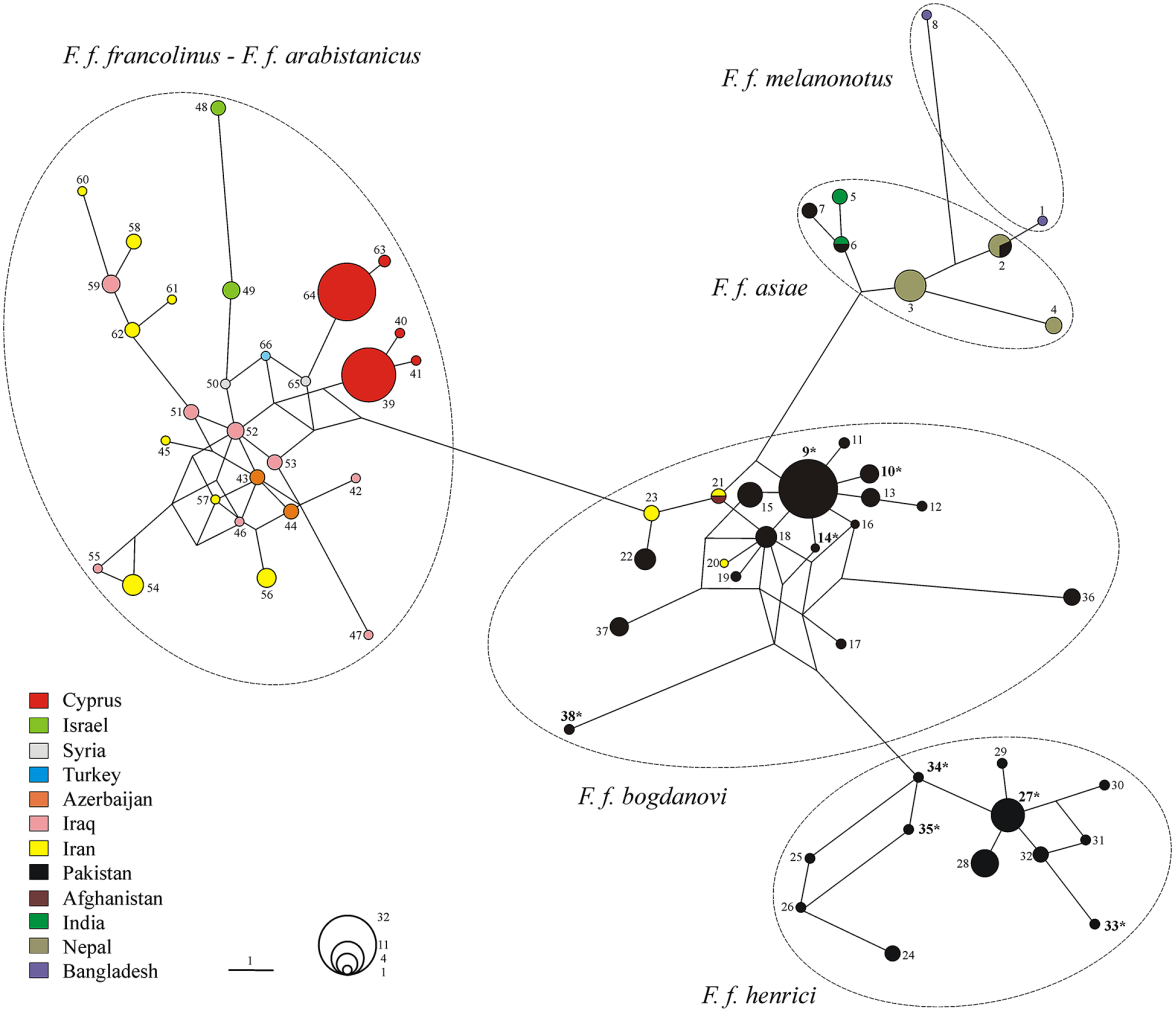
Black francolin haplotype network as computed with network. The size of circles corresponds to the number of haplotypes (H1 to H66) and the length of lines between them is proportional to the number of mutations. The colour of each country is indicated as well as the number of each haplotype (S1 Table). Black francolin subspecies are indicated as well. Haplotypes including new sequences with respect to the study of Forcina et al. [34] are reported in bold with an asterisk (H9, H10, H14, H27, H33-35 and H38).

The Median Joining network pointed to the occurrence of three major haplogroups (Fig 2, S1 Table). While no differentiation was found between *francolinus* and *arabistanicus* subspecies, the *bogdanovi—henrici* haplogroup included two clusters. The first, *F*. *f*. *bogdanovi*, comprised 63 individuals from Pakistan (North: 9; Central: 42; South: 12) and 5 from South East Iran and Afghanistan. The second, *F*. *f*. *henrici*, included 31 francolins from Pakistan only (North: 11; Central: 13; South: 7). Finally, the *asiae—melanonotus* haplogroup comprised four black francolins from Pakistan (North: 1; South: 3) and 19 from India, Nepal and Bangladesh. Overall, *F*. *f*. *bogdanovi* birds were separated from the *francolinus—arabistanicus* and *asiae—melanonotus* haplogroups by seven and six mutational steps, respectively, while five mutations stepped away the closest relatives of *F*. *f*. *bogdanovi* and *F*. *f*. *henrici*. Among the 23 Pakistani birds new to this study, 16 and 7 were assigned to *F*. *f*. *bogdanovi* (H9, H10, H14, H38) and *F*. *f*. *henrici* (H27, H33-H35), respectively. The population of Badin included haplotypes from all subspecies occurring in Pakistan (*F*. *f*. *bogdanovi*: H9, H16 and H18, *N* = 7; *F*. *f*. *henrici*: H24-H26 and H29-H31, *N* = 6; *F*. *f*. *asiae*: H6-H7, *N* = 3; Fig 2, Table 1, S1 Table).

**Table 1:** Number of individuals (all, males, females) assigned to *F*. *f*. *bogdanovi*, *F*. *f*. *henrici* and *F*. *f*. *asiae* in North, Central and South Pakistan according to the mtDNA network (Fig 2) and the Bayesian cluster analysis of STR multilocus genotypes (Fig 4).

		mtDNA			STR		
		All	Males	Females	All	Males	Females
North Pakistan *N* = 21	*F*. *f*. *bogdanovi*	9	8	1	2	2	0
	*F*. *f*. *henrici*	11	6	5	6	5	1
	*F*. *f*. *asiae*	1	1	0	4	0	4
	Admixed genotype	-	-	-	9	8	1
Central Pakistan *N* = 55	*F*. *f*. *bogdanovi*	42	27	15	19	13	6
	*F*. *f*. *henrici*	13	8	5	7	6	1
	*F*. *f*. *asiae*	0	0	0	1	1	0
	Admixed genotype	-	-	-	28	15	13
South Pakistan *N* = 22	*F*. *f*. *bogdanovi*	12	6	6	0	0	0
	*F*. *f*. *henrici*	7	6	1	5	4	1
	*F*. *f*. *asiae*	3	3	0	1	0	1
	Admixed genotype	-	-	-	16	11	5

Subspecies frequency distribution across Pakistan was reported in Table 1. While *F*. *f*. *bogdanovi* was the most abundant taxon in the Central region (76%), it decreased moving both northwards and southwards; *F*. *f*. *henrici* showed the lowest (23%) and highest (52%) occurrence in Central and North Pakistan, respectively. Finally, *F*. *f*. *asiae* (only male birds) was discovered in North (Kohala, H2, *N* = 1) and South (Badin, H6-H7, *N* = 3) Pakistan (Fig 2 and Table 1). Overall, all indexes indicated that North and South Pakistan hold the highest level of genetic diversity, although the highest number of haplotypes (n = 16) was found in the Central region (Table 2).

**Table 2:** Genetic diversity (mtDNA, STR) as inferred for North, Central, and South Pakistan black francolins (males + females; Fig 1).

	mtDNA					STR										
	*N*	*n*	*S*	*h*	*k*	*π* (%)	*n*A	*n*a	Ar		*I*_N_	*H*_O_	*H*_E_	*P*	Chi^2^ (d.f.)	*f*
North Pakistan	21	8	24	0.800	6.809	0.58	3.4	1.0	3.4		0.374	0.526	0.621	< 0.001*	∞ (14)	0.158
Central Pakistan	55	16	24	0.754	4.248	0.36	7.1	20.0	4.5		0.468	0.453	0.536	< 0.001*	∞ (18)	0.160
South Pakistan	22	14	20	0.900	6.563	0.56	4.6	3.0	4.1		0.445	0.446	0.535	< 0.001*	52.9 (18)	0.172

*N*, sample size; *n*, number of haplotypes; *S*, polymorphic sites; *h*, haplotype diversity; *k*, average numbers of nucleotide differences; π, nucleotide diversity; *n*A, number of alleles per locus; *n*a, number of unique alleles; A_r_, allelic richness; *I*_n_, Nei’s index *H*_O_: observed heterozygosity; *H*_E_, expected heterozygosity; *P*, probability value for HWE test; Chi^2^, test with relative degrees of freedom (d.f.) (Fisher global test, all loci); *f*, inbreeding coefficient.

*: significant departure from HWE

**Fig 3:**
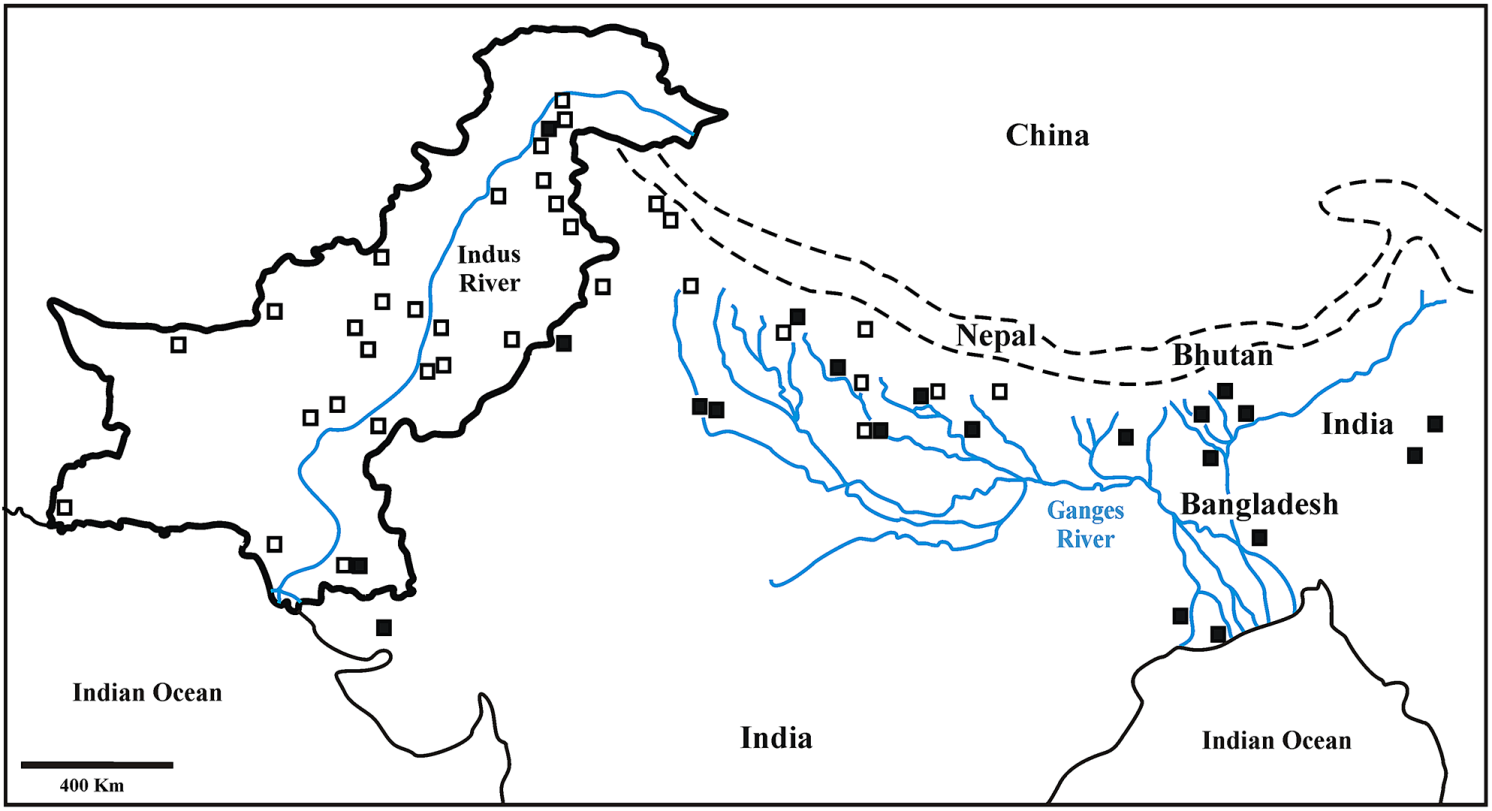
The distribution of black francolin subspecies across the Indian sub-continent as inferred from the 185-bp long mtDNA network including 298 modern + archival black francolins (see Results of this study and Forcina et al.) [29]. Localities with birds assigned to *bogdanovi*—*henrici* and *asiae*—*melanonotus* haplogroup are indicated by means of open and solid squares, respectively. Both Indus River and Ganges River Basin are reported in the map. Dashed lines mark out the main mountain range.

Despite the inclusion of 23 new birds, the 185 bp-long alignment of modern + archival francolins did not reveal any new haplotype (H1 to H72) with respect to those reported by Forcina et al. [29]. We created a subspecies distribution map and found that *F*. *f*. *bogdanovi* and *F*. *f*. *henrici* occur from Pakistan across northern India and Nepal towards Bangladesh with a decreasing gradient along the Ganges River Basin, whereas *F*. *f*. *asiae* and *F*. *f*. *melanonotus* follow the same trend but in the opposite direction (Fig 3).

### Genetic data repositories

Entire mtDNA CR alignment and STR genotyping data are available at TreeBASE (permanent link: http://purl.org/phylo/treebase/phylows/study/TB2:S22601), while GenBank accession codes (LT990153-57) are listed in S1 and S2 Tables.

### Microsatellite DNA analysis

The STR panel was powerful in discriminating individuals (*P*_ID_ = 7.4 x 10^−7^ and *P*_ID_sib = 3.8 x 10^−3^: S3 Table) [49] and all loci were polymorphic. No evidence for allele dropout and scoring errors was found, while the 8.5% of the microsatellite turned out to be null alleles. There was no evidence for LD after Bonferroni correction (*P* > 0.0014, all comparisons: S4 Table). The amova computed for North, Central and South Pakistan showed that 98.3% of the total STR variability was partitioned within regions and 1.7% among them (*F*_st_ = 0.02, *P* = 0.01).

**Fig 4:**
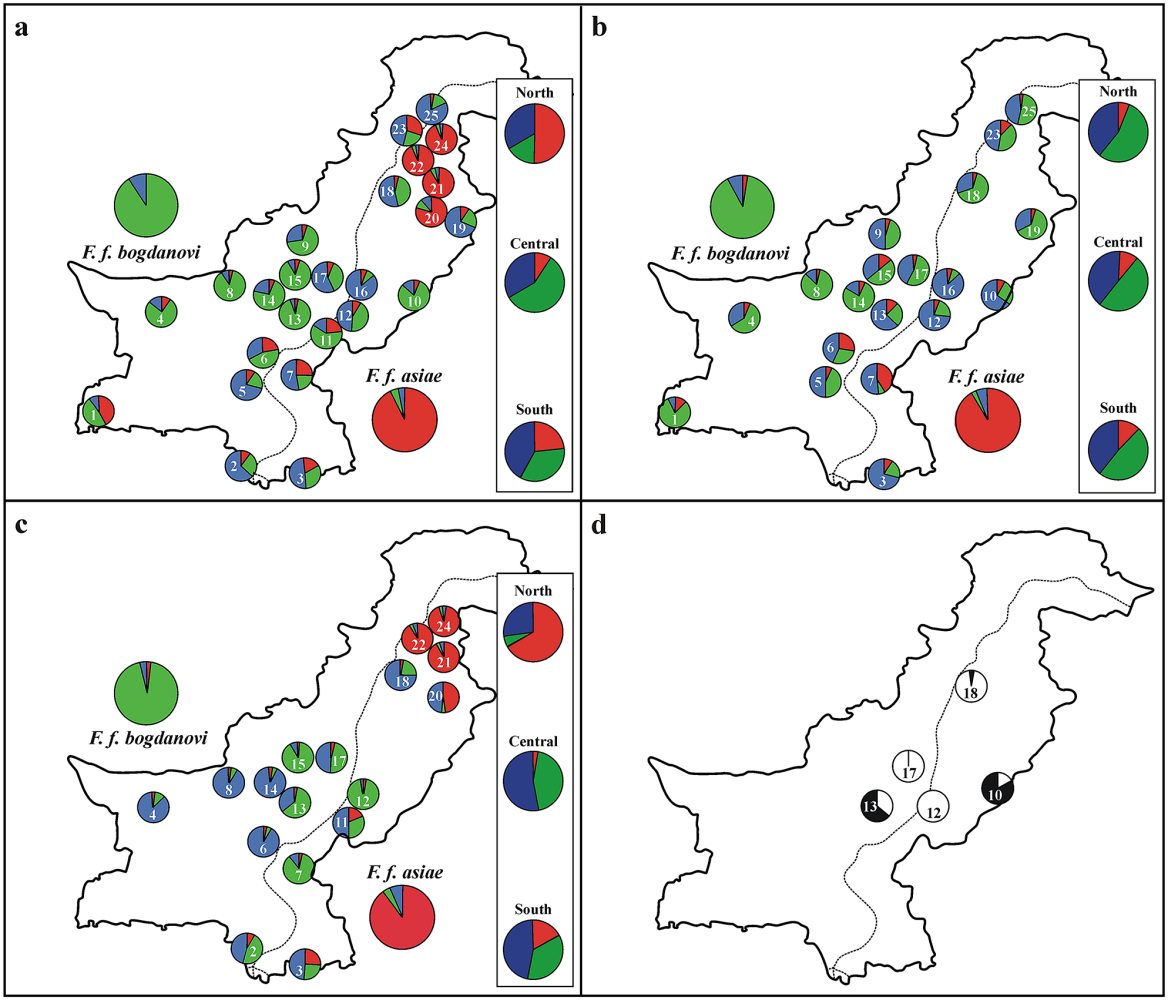
Bayesian analysis of STR multilocus genotypes as inferred with structure for each investigated population using (a) all individuals, (b) male and (c) female black francolins. Δ*K* was optimal with *K* = 3 for all computations. Genetic structure for both single (1 to 25) and grouped (North, Central, and South Pakistan) populations is given by means of a pie chart whose segments are proportional to the estimated membership to the *K* clusters (Fig 1, S1 Table). Individuals from Iran/Afghanistan and India/Nepal were included in the computations as genetic reference for *F*. *f*. *bogdanovi* (green) and *F*. *f*. *asiae* (red), respectively, while black francolins belonging to *F*. *f*. *henrici* genetic group are showed in blue. When populations are not included in panel b or c means that they do not harbour any male or female francolin, respectively. (d) Bayesian analysis of STR multilocus genotypes of the same populations investigated by Riaz et al. [23] using RAPDs (Haroon Abad, 10; Alipur, 12; Rakhni, 13; Bait Suvai, 17; Chakwal, 18). Birds from Alipur, Bait Suvai and Chakwal are assigned to the white cluster, while those from Haroon Abad and Rakhni to that in dark grey colour.

We found the highest values of genetic diversity in the Central region while the lowest ones occurred in North Pakistan (all indexes: Table 2). We observed significant departures from HWE due to heterozygote deficiency in North, Central and South Pakistan (Table 2, S5 Table) Bayesian clustering analysis performed with structure (all individuals, males and females only) pointed to the existence of three genetic groups (Fig 4, Table 1, S6 Table). Black francolins from Iran and Afghanistan and those from India and Nepal were assigned to two separated clusters (Iran and Afghanistan: Q_I_ range: 0.90–0.96; India and Nepal: Q_II_ range: 0.89–0.96; S6 Table), and they were used as *F*. *f*. *bogdanovi* (Fig 4: in green) and *F*. *f*. *asiae* (Fig 4: in red) subspecies genetic reference, respectively. Consequently, the third cluster (Fig 4: in blue) was deemed to include *F*. *f*. *henrici* representatives. Individuals (males + females) assigned to *F*. *f*. *bogdanovi* were the most abundant ones in the Central region, whereas they decreased moving both northwards and southwards (Table 1). Admixed genotypes (males + females) were widespread across both Central and South Pakistan, but males showed higher admixture values than females in all regions (Table 1). Populations (males + females) from North Pakistan showed a higher assignment value to *F*. *f*. *asiae*, which occurred also in the South (North, Q_II_: 0.51; South, Q_II_: 0.23; Fig 4a and S6 Table). This pattern was even more evident when females only were taken into account (North, Q_II_: 0.67; South, Q_II_: 0.17; Fig 4c, S6 Table), with four and one individuals assigned to *F*. *f*. *asiae* in North and South Pakistan, respectively (Table 1). Moreover, females with *F*. *f*. *bogdanovi* genotypes were absent in the North (Q_I_: 0.06) while they occurred in the Central region along with those with *F*. *f*. *henrici* genotypes (Q_I_: 0.44 and Q_III_: 0.52; Fig 4a and S6 Table). As far as the male individuals are concerned, admixed assignment values to the three subspecies were comparable across all regions (Q_I_ range: 0.41–0.55; Q_II_ range: 0.05–0.11; Q_III_ range: 0.38–0.48; Fig 4b, S6 Table), with birds allocated to *F*. *f*. *asiae* subspecies occurring also in Central Pakistan (Table 1).

Gene flow values computed among the North, Central and South Pakistan were lower in females than in males (average *N*_e_m, females = 1.03, males = 2.18: Table 3), with the highest values occurring between North and Central Pakistan and Central and South Pakistan (all comparisons, Table 3).

**Table 3:** STR-based gene flow estimates among North, Central, and South Pakistan black francolins (all, males and females).

	North Pakistan	Central Pakistan	South Pakistan
All individuals			
North Pakistan	-	3.11	1.73
Central Pakistan	-	-	2.99
South Pakistan	-	-	-
Males			
North Pakistan	-	2.83	1.11
Central Pakistan	-	-	2.61
South Pakistan	-	-	-
Females			
North Pakistan	-	1.10	0.71
Central Pakistan	-	-	1.27
South Pakistan	-	-	-

The second Bayesian clustering analysis was performed only for the black francolin (males + females) populations of Haroon Abad, Alipur, Rakhni, Bait Suvai and Chakwal. Results pointed to the occurrence of two genetic groups (Fig 4d, S4 Table): birds from Alipur, Bait Suvai and Chakwal were assigned to cluster Q_I_ (Q_I_ ≥ 0.99), while those from Haroon Abad and Rakhni showed their highest assignment values to cluster Q_II_ (0.83 and 0.65, respectively).

## Discussion

### Biogeography and diversity of the black francolin in Pakistan

Low-vagile species are expected to show well-structured phylogeographic patterns across their distribution ranges [59]. Most galliform species are sedentary (92%) [60], displaying high site fidelity and low dispersal ability [61], and the black francolin is not an exception [21]. Accordingly, the reappraisal of the species phylogeography with molecular tools [34] turned into a spatially well- marked picture consistent with the morphologic groups and their distribution as proposed since Dementiev [62].

In the present study, when only Pakistan was taken into account, our results delivered a more complex scenario. Genetic analyses did not show a clear spatial pattern of haplogroups in any of the subspecies (Fig 2), with their distribution displaying overt admixture zones from North to South Pakistan. The co-occurrence of multiple black francolin subspecies in Pakistan could be interpreted as the result of the faunal reshuffling following repeated cycles of wetland expansion and contraction in the Indian sub-continent since the mid-Miocene times. Indeed, fossil record indicates that in this period the region was covered with a dense humid forest stretching with no interruption to South East Asia, thus offering a wide ecological niche to trigger the diversification and dispersal of avian taxa from South East India to northwest and further. The late Miocene and Pliocene climatic oscillations turned into the progressive shrinking of large tracts of humid forest cover and its retreat to wet zones in India, followed by further cycles of expansion/contraction associated with the alternation of dry and wet periods [63]. During Pleistocene, the monsoon regime further intensified and, on the one hand, it contributed to establish large river systems subject to recurrent over-spilling and flooding. On the other hand, it triggered the progressive drying up of the Indian subcontinent [64]. These fluctuations are at the basis of the marked discontinuity in the distribution of many wet-zone species typical to South Asian biogeography [65], especially birds, mammals, reptiles and other components of the Indian sub-continent biota [63]. By providing a composite assemblage of habitats, these shifts also boosted adaptive radiations in the lowlands as opposed to the nearby Himalayan region, where spatial and trophic niches had already reached saturation turning into slowing down of the diversification rate [66–69]. Overall, home to three of the six morphological subspecies and previously evidenced as the ancestral area for the westward *F*. *francolinus* adaptive diversification [34], which, in turn, fits with the direction of most avian radiations across the Palaearctic [70], the Indian sub-continent played as a crucial pathway for the species biogeography.

**Fig 5:**
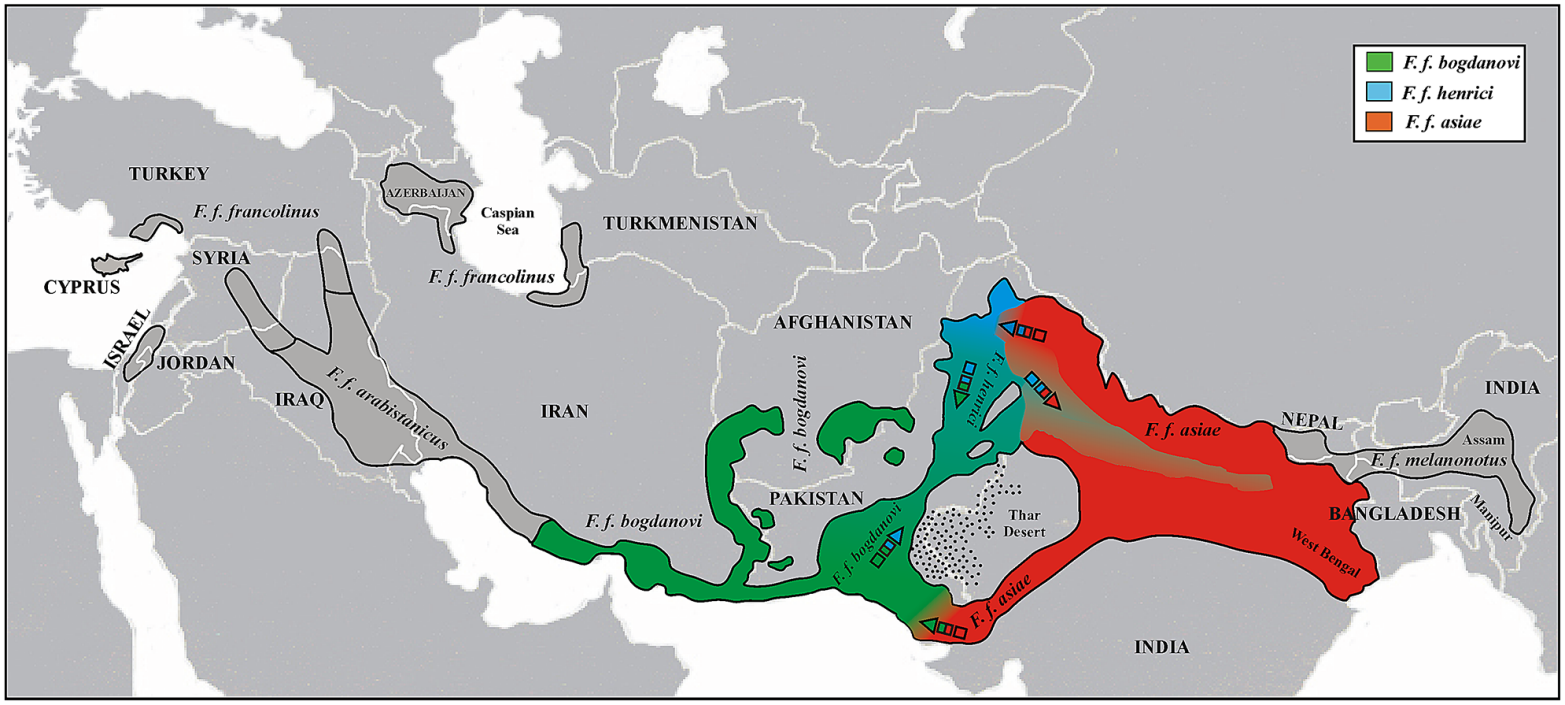
Distribution range of *F*. *francolinus* (black continuous line) with subspecies boundaries (dotted lines). The ranges of the taxa included in this study are given in colour. Admixture zones inferred with genetic data are represented by intermediate shades. Arrows indicate (re)colonisation routes.

Mitochondrial DNA data pointed to *F*. *f*. *bogdanovi* and *F*. *f*. *henrici* as the most abundant subspecies in Central and North Pakistan, respectively, while confirming the *F*. *f*. *asiae* as likely relict in both the extreme North and South of the country [34]. Concordantly, these regions hosted the highest haplotype diversity value and represented the main gateways for Indian faunal assemblages in Pakistan. Whereas *F*. *f*. *asiae* presumably entered North Pakistan from the bordering Indian Kashmir, it thrived in the southern part of the country as relict of Indian fauna within the Pakistani range of The Great Rann of Kachchh [34]. Moreover, the highest number of haplotypes and the seemingly discordant lowest values of genetic diversity indexes observed in Central Pakistan (Table 2) would suggest this area to have played as source for (re)colonization warranting eventually higher diversity elsewhere [71]. Indeed, when considering the distribution of black francolin subspecies in the whole Indian sub-continent (Fig 3), individuals clustering into *henrici-bogdanovi* haplogroup were found from Pakistan eastwards across northern India to Nepal along the Ganges River Basin. This result is in agreement with Whistler [72], who, unlike other authors [17–20, 73,74] reported *F*. *f*. *henrici* for the same areas, and might suggests a *F*. *f*. *henrici* backward recolonisation along the Ganges River Basin following *F*. *f*. *asiae* local extinction. This assumption also relies on the disclosure of isolated representatives of *F*. *f*. *asiae* in North Pakistan as well as in India both north and south to *F*. *f*. *henrici* distribution (Fig 3), and the pattern of subspecies divergence in the context of the westward adaptive diversification of the species [34]. Moreover, it is entirely consistent with the local recurrent regression of wetlands described by Karanth [63]. Accordingly, evidence for both southward and northward population expansion along the Indus River Valley had already emerged in Pakistan [34]. Therefore, the overall scenario points to the occurrence of two crucial geographic frameworks for the adaptive diversification of the black francolin throughout the Indian sub-continent corresponding to its major rivers and their reaches, the Indus River Valley and the Ganges River Basin (Fig 5). This complex picture of population expansion, local extinction and recolonisation turning into areas of high diversity is corroborated by the partition of genetic variability that was significantly higher within rather than among geographic areas at both the mitochondrial (89.05%: *ϕ*_st_ = 0.11, *P* < 0.001) and nuclear (98.3%: *F*_st_ = 0.02, *P* = 0.01) DNA. Accordingly, STR-based gene flow estimates (Table 3) pointed to a substantial exchange between North and Central Pakistan as well as Central and South Pakistan.

Nevertheless, while also indicating *F*. *f*. *bogdanovi* as the dominant subspecies in Central Pakistan (Fig 4), microsatellite data showed the highest genetic diversity values (number of private alleles included) therein and the lowest in the North (Table 2), in sharp contrast with the picture inferred from mitochondrial DNA. Likewise, genetic admixture among subspecies was found to be higher in Central Pakistan, pointing to it as a sink area, while the highest assignment values to *F*. *f*. *asiae* in the North and the South would rather point to these regions as source areas, with *F*. *f*. *bogdanovi* presumably conveying the genetic mixing among different subspecies. However, when subspecies assignment and genetic mixing were examined across regions and according to sex, we found a similar pattern, with by far more admixed males than females. We rule out this outcome to be somehow biased by the uneven sex *ratio* of our sample including twice as many males than females (S1 Table). Among phasianids, sex *ratio* is often skewed in favour of males, possibly as a consequence of the higher mortality experienced by females during incubation [75, 76]. Some authors even speculated upon a routinely higher natural female mortality for heterogametic (ZW) sex in birds [77]. However, with a male/female sex *ratio* of 1.31 [26], black francolins from Pakistan are not an exception. Moreover, when the analyses were repeated on only the female sample, mitochondrial and nuclear data found no *F*. *f*. *asiae* and almost no *F*. *f*. *bogdanovi* representatives in Central and North Pakistan, respectively (Fig 4, Table 1). Interestingly, the North was indicated as the core of *F*. *f*. *asiae* distribution in the country by the higher assignment values of both the sample as a whole and, even more, the female one (Fig 4a, S6 Table). Conversely, when only males were considered, the highly admixed scenario with *F*. *f*. *asiae* found everywhere and the other subspecies evenly distributed would indicate a higher dispersal by males. In addition, since most avian species show a philopatric male structure [78], we would eventually expect the female population to be more heterogeneous when compared to the less dispersing male one. Nevertheless, our results indicate the opposite, with the gene flow among regions being much lower in females than in males (Table 3) and locally higher population assignment values in the female sample contrasting with the lower values evenly distributed across regions in the male sample (Fig 4b, S6 Table). Among the generally sedentary and low vagile phasianids a similar scenario is unlikely, and may occur with a few exceptions in species exhibiting higher mobility and female social or genetic sequential polygamy. As case in point, common quail (*Coturnix coturnix*) males, which provide no parental cares, migrate to higher latitude breeding grounds first, mate, and move further in the search of new partners, while females reside in the same location for the whole breeding season [79]. However, in the monogamous black francolin, females produce two clutches per season and both parents rear the chicks [18].

To conclude, the partly discordant picture retrieved with different markers might be indicative of different phenomena shaping the genetic diversity of the black francolin populations in Pakistan. While the maternally inherited mitochondrial DNA delivered a composite assemblage of subspecies whose pattern of co-occurrence is compatible with the geological history and the faunal movement routes of the region under study, the biparentally inherited microsatellite portrayed an extensive, male-biased genetic mixing demanding for further explanations. These somehow puzzling results most likely points towards the impact of human-mediated relocations of male black francolins in Pakistan.

### Management issues

Different to most of the nearby countries, Pakistan allows hunting of the black francolin. With their bright plumage and calls from prominent posts heard miles away, males are a particularly easy target for hunters. However, living males are also highly demanded and traded as pets for the chirping competitions [23]. Forbidden in India, this practice is still common in Afghanistan and Pakistan, where sell and buy of black francolins is extensive, with purchase price ranging from 600 to 2,000 US$ per individual. Translocated males are released intentionally or accidentally into the wild across different regions and can alter the genetic structure of local black francolin populations. Indeed, when only females are taken into account, the pattern of subspecies distribution in Pakistan is even clearer probably because it is not heavily blurred by the effects of restocking. In turn, when the male dataset only was taken into account, no female counterparts occurred for some sampling localities (overall, 8 out of 25 sampling localities: S1 Table) or it alone accounted for the presence of a subspecies in a given area, and precisely *F*. *f*. *asiae* in Central Pakistan. Interestingly, the latter is apparently the most commercialized among the black francolin subspecies, as indicated by introductions to US [80], mainland Europe [17, 81] and, more recently, by translocations to Cyprus [46], thus calling for its yet limited occurrence in Central Pakistan as another such case. It is indicative that the districts hosting the liveliest chirping competitions are primarily in Central (Bhakkar and Mianwali: Punjab Province) and North (Bannu, Dera Ismail Khan, Kohat, Lakki Marwat and Peshawar: Khyber Pakhtunkhwa Province) Pakistan—precisely where the genetic mixing turned out to be higher (A.A. Khan, personal communication, 2018).

Noteworthy, the human-mediated genetic mixing in Pakistani black francolin invoked by Riaz et al. [23] was ultimately confirmed. Multilocus STR genotypes of samples from the same localities investigated with RAPDs by these authors showed a similar counter-intuitive increase of genetic similarity between geographically more distant populations (e.g., Haroon Abad and Rakhni, Fig 4d; see also Fig 1). Such an extensive biotic homogenization raises serious concern for the long-term preservation of the black francolin in Pakistan as elsewhere, in that it may erase locally adapted genotypes and lessen the evolutionary potential in face of increasing global change and human disturbance [82]. The introduction of nonnative genotypes often determines the loss of the genetic identity in association with the introduction of maladaptive alleles [83] and decrease in diversity [7, 84] potentially turning into outbreeding depression [85]. The spreading of maladaptive traits in local avian population through releases of nonnative stocks and consequent introgression has been widely documented in the red-legged partridge [86] and in the common quail [15]. This process, which may eventually involve detrimental changes in behaviour and pathogen spread [87], has been proven to occur even between conspecific populations [8], game birds included [11]. At the moment no such data are available for the black francolin, nor is possible to rule out *a priori* the possibility that this unintentional genetic mixing might play some sort of genetic rescue effect on declining populations [88]. Nevertheless, translocations of philopatric avian species were showed to be successful on condition that mostly immatures are released [89–90], this not being the case of the present study. On the other hand, the heterozygote deficiency we detected could be possibly indicative of ongoing drift and, as such, suggest that either (i) translocations are hastening the decline by erasing genetic diversity or (ii) that no genetic rescue effect is occurring at all and that (iii), if any, it is overridden by a likely demographic decrease. The ultimate impact of ongoing human-mediated hybridisation in Pakistani black francolin should be further investigated by monitoring the adaptive genetic diversity through time other than by means of complementary ecological studies. In the absence of more detailed information, we recommend the Pakistani law enforcement authorities to monitor and limit the uncontrolled sell and buy of these valuable birds, and to promote educational programs to foster the public awareness of the importance of preserving local biodiversity resources. Initiatives such as the organization of round tables, press releases, lectures in schools as well as training *ad hoc* for university students would help triggering the cultural change needed.

## Supporting information

S1 Table Sample size including Pakistan and other countries. Pakistani samples are listed per region (North, Central and South) including information on locality with (i) latitude (Lat.) and longitude (Long.) data, number (*N*) of individuals (male, M; female, F), (iii) sampled tissue, (iv) year(s) of sampling and (v) related CR mtDNA haplotype (cf., Fig 2). Samples collected outside Pakistan are those employed in the study of Forcina et al. [34] and are given as total number per each country with CR mtDNA haplotype assigned in the present study.
(PDF)

S2 Table GenBank accession codes. GenBank accession codes for the mtDNA CR haplotypes (H; entire gene length) used in the analyses of this study (1 to 66: see also S1 Table) and including those from the study of Forcina et al. [34].
(PDF)

S3 Table Multi-locus *P*_ID_ and *P*_ID_sib. Multi-locus *P*_ID_ (i.e., the probability that two individuals drawn at random share identical genotypes) and *P*_ID_sib (i.e., the probability of identity among siblings) data are provided. Loci are sorted according to the increasing order of their *P*_ID_ and *P*_ID_sib single-locus values (i.e., the locus at the top is the most informative one), and a sequentially multi-loci *P*_ID_ (*P*_ID_sib) is reported for each locus. See Forcina et al. [46] for details on the loci employed in the present study.
(PDF)

S4 Table Linkage Disequilibrium test. Fisher global test for departure from Linkage Disequilibrium (LD) for each pair of loci across all populations. No comparison was significant (Bonferroni correction: α = 0.05, α’ = α/36 = 0.0014). Legend: *P*, probability value for LD test; Chi^2^, test with relative degrees of freedom (d.f.) (Fisher’s method).
(PDF)

S5 Table Departure from Hardy-Weinberg Equilibrium test. Observed (*H*_O_) and expected (*H*_E_) heterozygosity as inferred for North, Central, and South Pakistan black francolins. The probability value for HWE test (*P*) was calculated for each locus. *: significant departure from HWE after application of Bonferroni correction (α = 0.05, α' = α/8 = 0.006). Mono., monomorphic locus.
(PDF)

S6 Table Posterior probability values from Bayesian analysis. Posterior probability of membership to each of the three clusters as inferred by structure for (i) all single populations (with number, see Fig 1 and S1 Table), (ii) North, Central and South Pakistan, and (iii) *F*. *f*. *bogdanovi* and *F*. *f*. *asiae* genetic references (Fig 4). Subspecies assignment: Q_I_, *F*. *f*. *bogdanovi*; Q_II_, *F*. *f*. *asiae*; Q_III_, *F*. *f*. *henrici* (Fig 4: green, red and blue, respectively).
(PDF)
