# Supplementary material for: Human-modified biogeographic patterns and conservation in game birds: The dilemma of the black francolin (Francolinus francolinus, Phasianidae) in Pakistan
Source: PLoS One. 2018 Oct 5;13(10):e0205059. doi: 10.1371/journal.pone.0205059 (PMC6173408; doi:10.1371/journal.pone.0205059)
Supplement: S1 Table — Pakistani samples are listed per region (North, Central and South) including information on locality with (i) latitude (Lat.) and longitude (Long.) data, number (N) of individuals (male, M; female, F), (iii) sampled tissue, (iv) year(s) of sampling and (v) related CR mtDNA haplotype (cf., Fig 2). Samples collected outside Pakistan are those employed in the study of Forcina et al. [34] and are given as total number per each country with CR mtDNA haplotype assigned in the present study. (PDF) [file pone.0205059.s001.pdf]

S1 Table. **Sample size including Pakistan and other countries**. Pakistani samples are listed per region (North, Central and South) including information on locality with (i) latitude (Lat.) and longitude (Long.) data, number (*N*) of individuals (male, M; female, F), (iii) sampled tissue, (iv) year(s) of sampling and (v) related CR mtDNA haplotype (cf., Fig 2). Samples collected outside Pakistan are those employed in the study of Forcina et al. [34] and are given as total number per each country with CR mtDNA haplotype assigned in the present study.

**Country**	**Locality**	**Lat. N/Long. E**	***N* (M; F)**	**Tissue**	**Year(s)**	**Haplotype number**
**Pakistan**						
South						
Baluchistan	Dasht River, near Jiwani (1)	25°03'N; 61°45'E	2 (2; 0)	Feather	2009	H36
Sindh	Near Karachi (2)	24°51'N; 67°00'E	4 (0; 4)	Feather	2010	H9, H13, H28
Sindh	Badin (3)	24°39'N; 68°50'E	16 (13; 3)	Feather	2009	H6, H7, H9, H16, H18, H24-H26 H29-H31
Central						
Baluchistan	Chagai (4)	29°29'N; 64°70'E	3 (2; 1)	Feather	2014	H9
Sindh	Near Larkana (5)	27°55'N; 68°22'E	5 (5; 0)	Feather	2010	H9, H18, H28
Sindh	Jacobabad (6)	28°17'N; 68°26'E	3 (2; 1)	Feather	2009, 2013	H9, H14, H24
Sindh	Ghotki (7)	28°01'N; 69°19'E	4 (2; 2)	Feather	2008	H9, H35
Baluchistan	Quetta (8)	30°18'N; 66°99'E	3 (2; 1)	Feather	2014	H9, H33
Baluchistan	Zhoab (9)	31°34'N; 69°45'E	2 (2; 0)	Feather	2014	H9, H33
Punjab, Bahawalnagar	Haroon Abad (10)	29°37'N; 73°08'E	5 (5; 0)	Feather	2008	H15
Punjab, Muzaffar Garh	Basti Makwal (11)	30°07'N; 71°18'E	6 (0; 6)	Feather	2013	H10, H27, H34, H38
Punjab, Muzaffar Garh	Alipur (12)	29°23'N; 70°18'E	3 (2; 1)	Feather	2008	H9, H10
Baluchistan, Barkhan	Rakhni (13)	30°03'N; 69°55'E	2 (1; 1)	Feather	2008	H9, H27
Baluchistan, Loralai	Mekhtar (14)	30°41'N; 68°59'E	2 (1; 1)	Feather	2013	H9
Baluchistan, Musa Khel	Musa Khel (15)	30°52'N; 69°49'E	3 (2; 1)	Feather	2013, 2014	H9
Punjab, Muzaffar Garh	Ghazi Ghat (16)	30°06'N; 70°80'E	1 (1; 0)	Feather	2008	H9
Punjab, Dera Ghazi Khan	Bait Suvai (17)	30°45'N; 70°89'E	13 (8; 5)	Feather	2008	H9, H12, H13, H17, H28, H32
North						
Punjab, Chakwal	Rabal (18)	32°56'N; 72°52'E	6 (4; 2)	Feather	2008	H27
Punjab, Sialkot	Sialkot (19)	32°35'N; 74°30'E	6 (6; 0)	Feather	2009	H9, H11, H19, H22, H32
Azad Jammu Kashmir	Bhimber (20)	32°58'N; 74°02'E	1 (0; 1)	Feather	2009	H28
Azad Jammu Kashmir	Mirpur (21)	33°08'N; 73°44'E	1 (0; 1)	Feather	2009	H27
Azad Jammu Kashmir	Nikyal, near Kotli (22)	33°28'N; 74°06'E	1 (0; 1)	Feather	2008	H22
Azad Jammu Kashmir	Kohala (23)	34°12'N; 73°29'E	3 (3; 0)	Feather	2009	H2, H27, H37
Azad Jammu Kashmir	Tandali (24)	34°13'N; 73°29'E	1 (0; 1)	Feather	2009	H27
Azad Jammu Kashmir	Muzaffarabad (25)	34°22'N; 73°28'E	2 (2; 0)	Feather	2009	H37
**Total**			**98 (65; 33)**			
**Other countries**						
Afghanistan	-	-	1	Feather	2010	H21
Azerbaijan	-	-	4	Feather	2011	H43, H44
Bangladesh	-	-	2	Feather	2009	H1, H8
Cyprus	-	-	59	Liver	2007-2011	H39, H40, H41, H63, H64
India	-	-	3	Feather	2011	H5, H6
Iran	-	-	19	Feather	2009-2011	H20, H21, H23, H45, H54, H56-H58, H60-H62
Iraq	-	-	14	Feather	2009, 2010	H42, H46, H47, H51-H53, H55 H59
Israel	-	-	5	Feather	2007-2009	H48, H49
Nepal	-	-	14	Feather	2008	H2-H4
Syria	-	-	2	Feather	2010	H50, H65
Turkey	-	-	1	Feather	2010	H66
**Total**			**124**			
**Grand total**			**222**			
