# Supplementary material for: Human-modified biogeographic patterns and conservation in game birds: The dilemma of the black francolin (Francolinus francolinus, Phasianidae) in Pakistan
Source: PLoS One. 2018 Oct 5;13(10):e0205059. doi: 10.1371/journal.pone.0205059 (PMC6173408; doi:10.1371/journal.pone.0205059)
Supplement: S2 Table — GenBank accession codes for the mtDNA CR haplotypes (H; entire gene length) used in the analyses of this study (1 to 66: see also S1 Table) and including those from the study of Forcina et al. [34]. (PDF) [file pone.0205059.s002.pdf]

**S2 Table**. **GenBank accession codes**. GenBank accession codes for the mtDNA CR haplotypes (H; entire gene length) used in the analyses of this study (1 to 66: see also S1 Table) and including those from the study of Forcina et al. [34].

| Haplotype | Accession code | Literature record |
|---|---|---|
| H1 | HE793484 | Forcina et al. [34] |
| H2 | HE793485 | Forcina et al. [34] |
| H3 | HE793486 | Forcina et al. [34] |
| H4 | HE793487 | Forcina et al. [34] |
| H5 | HE793488 | Forcina et al. [34] |
| H6 | HE793489 | Forcina et al. [34] |
| H7 | HE793490 | Forcina et al. [34] |
| H8 | HE793491 | Forcina et al. [34] |
| H9 | HE793459 | Forcina et al. [34] |
| H10 | HE793460 | Forcina et al. [34] |
| H11 | HE793461 | Forcina et al. [34] |
| H12 | HE793463 | Forcina et al. [34] |
| H13 | HE793462 | Forcina et al. [34] |
| H14 | LT990153 | This study |
| H15 | HE793469 | Forcina et al. [34] |
| H16 | HE793464 | Forcina et al. [34] |
| H17 | HE793465 | Forcina et al. [34] |
| H18 | HE793466 | Forcina et al. [34] |
| H19 | HE793467 | Forcina et al. [34] |
| H20 | HE793468 | Forcina et al. [34] |
| H21 | HE793470 | Forcina et al. [34] |
| H22 | HE793483 | Forcina et al. [34] |
| H23 | HE793482 | Forcina et al. [34] |
| H24 | HE793473 | Forcina et al. [34] |
| H25 | HE793471 | Forcina et al. [34] |
| H26 | HE793472 | Forcina et al. [34] |
| H27 | HE793475 | Forcina et al. [34] |
| H28 | HE793474 | Forcina et al. [34] |
| H29 | HE793476 | Forcina et al. [34] |
| H30 | HE793477 | Forcina et al. [34] |
| H31 | HE793478 | Forcina et al. [34] |
| H32 | HE793479 | Forcina et al. [34] |
| H33 | LT990154 | This study |
| H34 | LT990155 | This study |
| H35 | LT990156 | This study |
| H36 | HE793480 | Forcina et al. [34] |
| H37 | HE793481 | Forcina et al. [34] |
| H38 | LT990157 | This study |
| H39 | HE793456 | Forcina et al. [34] |
| H40 | HE793457 | Forcina et al. [34] |
| H41 | HE793458 | Forcina et al. [34] |
| H42 | HE793432 | Forcina et al. [34] |
| H43 | HE793431 | Forcina et al. [34] |
| H44 | HE793439 | Forcina et al. [34] |
| H45 | HE793433 | Forcina et al. [34] |
| H46 | HE793438 | Forcina et al. [34] |
| H47 | HE793447 | Forcina et al. [34] |
| H48 | HE793444 | Forcina et al. [34] |
| H49 | HE793445 | Forcina et al. [34] |
| H50 | HE793446 | Forcina et al. [34] |
| H51 | HE793449 | Forcina et al. [34] |
| H52 | HE793450 | Forcina et al. [34] |
| H53 | HE793448 | Forcina et al. [34] |
| H54 | HE793434 | Forcina et al. [34] |
| H55 | HE793435 | Forcina et al. [34] |
| H56 | HE793436 | Forcina et al. [34] |
| H57 | HE793437 | Forcina et al. [34] |
| H58 | HE793451 | Forcina et al. [34] |
| H59 | HE793452 | Forcina et al. [34] |
| H60 | HE793453 | Forcina et al. [34] |
| H61 | HE793454 | Forcina et al. [34] |
| H62 | HE793455 | Forcina et al. [34] |
| H63 | HE793441 | Forcina et al. [34] |
| H64 | HE793440 | Forcina et al. [34] |
| H65 | HE793442 | Forcina et al. [34] |
| H66 | HE793443 | Forcina et al. [34] |
