# Supplementary material for: Human-modified biogeographic patterns and conservation in game birds: The dilemma of the black francolin (Francolinus francolinus, Phasianidae) in Pakistan
Source: PLoS One. 2018 Oct 5;13(10):e0205059. doi: 10.1371/journal.pone.0205059 (PMC6173408; doi:10.1371/journal.pone.0205059)
Supplement: S3 Table — Multi-locus PID (i.e., the probability that two individuals drawn at random share identical genotypes) and PIDsib (i.e., the probability of identity among siblings) data are provided. Loci are sorted according to the increasing order of their PID and PIDsib single-locus values (i.e., the locus at the top is the most informative one), and a sequentially multi-loci PID (PIDsib) is reported for each locus. See Forcina et al. [46] for details on the loci employed in the present study. (PDF) [file pone.0205059.s003.pdf]

S3 Table. **Multi-locus P_{ID} and P_{ID}sib.** Multi-locus P_{ID} (i.e., the probability that two individuals drawn at random share identical genotypes) and P_{ID}sib (i.e., the probability of identity among siblings) data are provided. Loci are sorted according to the increasing order of their P_{ID} and P_{ID}sib single-locus values (i.e., the locus at the top is the most informative one), and a sequentially multi-loci P_{ID} (P_{ID}sib) is reported for each locus. See Forcina et al. [46] for details on the loci employed in the present study.

**Locus**	P_{ID}	P_{ID}**sib**
MCW 252	1.2×10^{-2}	2.9×10^{-1}
MCW 212	1.3×10^{-3}	1.2×10^{-1}
MCW 104	2.2×10^{-4}	5.5×10^{-2}
MCW 146	4.9×10^{-5}	2.8×10^{-2}
MCW 295	1.2×10^{-5}	1.4×10^{-2}
Aru 1.23	4.1×10^{-6}	8.5×10^{-3}
MCW 127	1.5×10^{-6}	5.4×10^{-3}
LEI 30	8.6×10^{-7}	4.1×10^{-3}
MCW 280	7.4×10^{-7}	3.8×10^{-3}
