# Supplementary material for: Human-modified biogeographic patterns and conservation in game birds: The dilemma of the black francolin (Francolinus francolinus, Phasianidae) in Pakistan
Source: PLoS One. 2018 Oct 5;13(10):e0205059. doi: 10.1371/journal.pone.0205059 (PMC6173408; doi:10.1371/journal.pone.0205059)
Supplement: S4 Table — Fisher global test for departure from Linkage Disequilibrium (LD) for each pair of loci across all populations. No comparison was significant (Bonferroni correction: α = 0.05, α’ = α/36 = 0.0014). Legend: P, probability value for LD test; Chi2, test with relative degrees of freedom (d.f.) (Fisher’s method). (PDF) [file pone.0205059.s004.pdf]

**S4 Table**. **Linkage Disequilibrium test**. Fisher global test for departure from Linkage Disequilibrium (LD) for each pair of loci across all populations. No comparison was significant (Bonferroni correction: $\alpha = 0.05$, $\alpha' = \alpha/36 = 0.0014$). Legend: *P*, probability value for LD test; $Chi^2$, test with relative degrees of freedom (d.f.) (Fisher's method).

| **Loci** | **$Chi^2$** | **d.f.** | ***P*** |
|---|---|---|---|
| MCW 252 & MCW 104 | 20.83 | 6 | 0.002 |
| MCW 252 & Aru 1.23 | 3.18 | 6 | 0.786 |
| MCW 104 & Aru 1.23 | 6.59 | 6 | 0.359 |
| MCW 252 & MCW 146 | 12.02 | 6 | 0.061 |
| MCW 104 & MCW 146 | 14.36 | 6 | 0.026 |
| Aru 1.23 & MCW 146 | 10.64 | 6 | 0.100 |
| MCW 252 & MCW 212 | 23.93 | 6 | 0.002 |
| MCW 104 & MCW 212 | 9.70 | 6 | 0.138 |
| Aru 1.23 & MCW 212 | 7.93 | 6 | 0.243 |
| MCW 146 & MCW 212 | 7.57 | 6 | 0.271 |
| MCW 252 & MCW 295 | 20.33 | 6 | 0.002 |
| MCW 104 & MCW 295 | 9.90 | 6 | 0.129 |
| Aru 1.23 & MCW 295 | 5.56 | 6 | 0.473 |
| MCW 146 & MCW 295 | 21.92 | 6 | 0.002 |
| MCW 212 & MCW 295 | 3.82 | 6 | 0.702 |
| MCW 252 & MCW 127 | 9.16 | 6 | 0.165 |
| MCW 104 & MCW 127 | 15.05 | 6 | 0.020 |
| Aru 1.23 & MCW 127 | 5.35 | 6 | 0.499 |
| MCW 146 & MCW 127 | 12.37 | 6 | 0.054 |
| MCW 212 & MCW 127 | 10.78 | 6 | 0.095 |
| MCW 295 & MCW 127 | 7.18 | 6 | 0.305 |
| MCW 252 & LEI 30 | 5.83 | 4 | 0.212 |
| MCW 104 & LEI 30 | 2.62 | 4 | 0.624 |
| Aru 1.23 & LEI 30 | 1.87 | 4 | 0.760 |
| MCW 146 & LEI 30 | 9.27 | 4 | 0.055 |
| MCW 212 & LEI 30 | 3.09 | 4 | 0.543 |
| MCW 295 & LEI 30 | 6.68 | 4 | 0.154 |
| MCW 127 & LEI 30 | 2.11 | 4 | 0.715 |
| MCW 252 & MCW 280 | 5.29 | 4 | 0.258 |
| MCW 104 & MCW 280 | 0.27 | 4 | 0.992 |
| Aru 1.23 & MCW 280 | 1.39 | 4 | 0.846 |
| MCW 146 & MCW 280 | 2.56 | 4 | 0.633 |
| MCW 212 & MCW 280 | 2.54 | 4 | 0.637 |
| MCW 295 & MCW 280 | 3.85 | 4 | 0.425 |
| MCW 127 & MCW 280 | 5.58 | 4 | 0.233 |
| LEI 30 & MCW 280 | 7.69 | 4 | 0.104 |
| MCW 252 & MCW 104 | 20.83 | 6 | 0.002 |
| MCW 252 & Aru 1.23 | 3.18 | 6 | 0.786 |
| MCW 104 & Aru 1.23 | 6.59 | 6 | 0.359 |
| MCW 252  & MCW 146 | 12.02 | 6 | 0.061 |
| MCW 104 & MCW 146 | 14.36 | 6 | 0.026 |
