# Supplementary material for: Human-modified biogeographic patterns and conservation in game birds: The dilemma of the black francolin (Francolinus francolinus, Phasianidae) in Pakistan
Source: PLoS One. 2018 Oct 5;13(10):e0205059. doi: 10.1371/journal.pone.0205059 (PMC6173408; doi:10.1371/journal.pone.0205059)
Supplement: S5 Table — Observed (HO) and expected (HE) heterozygosity as inferred for North, Central, and South Pakistan black francolins. The probability value for HWE test (P) was calculated for each locus. *: significant departure from HWE after application of Bonferroni correction (α = 0.05, α' = α/8 = 0.006). Mono., monomorphic locus. (PDF) [file pone.0205059.s005.pdf]

S5 Table. **Departure from Hardy-Weinberg Equilibrium test**. Observed (H_O) and expected (H_E) heterozygosity as inferred for North, Central, and South Pakistan black francolins. The probability value for HWE test (P) was calculated for each locus. *: significant departure from HWE after application of Bonferroni correction (α'= 0.05, α' = α/8= 0.006). Mono., monomorphic locus.

**Locus**	**North Pakistan**		**Central Pakistan**		**South Pakistan**	
	H_O/H_E	P	H_O/H_E	P	H_O/H_E	P
MCW252	0.611/0.894	< 0.001*	0.784/0.908	0.001*	0.765/0.925	0.006
MCW 104	0.474/0.602	0.022	0.712/0.707	0.504	0.524/0.591	0.876
Aru 1.23	0.636/0.593	0.292	0.537/0.438	0.003	0.471/0.519	0.164
MCW 146	0.571/0.557	0.082	0.600/0.606	0.773	0.727/0.659	0.011
MCW 212	0.650/0.717	0.009	0.547/0.766	< 0.001*	0.476/0.664	0.013
MCW 295	0.619/0.596	0.041	0.491/0.579	< 0.001*	0.318/0.601	0.001*
MCW 127	0.118/0.392	0.002*	0.160/0.409	< 0.001*	0.350/0.424	0.082
LEI 30	-	Mono.	0.167/0.334	< 0.001*	0.238/0.296	0.448
MCW 280	-	Mono.	0.077/0.075	1.000	0.143/0.136	1.000
