# Supplementary material for: Human-modified biogeographic patterns and conservation in game birds: The dilemma of the black francolin (Francolinus francolinus, Phasianidae) in Pakistan
Source: PLoS One. 2018 Oct 5;13(10):e0205059. doi: 10.1371/journal.pone.0205059 (PMC6173408; doi:10.1371/journal.pone.0205059)
Supplement: S6 Table — Posterior probability of membership to each of the three clusters as inferred by structure for (i) all single populations (with number, see Fig 1 and S1 Table), (ii) North, Central and South Pakistan, and (iii) F. f. bogdanovi and F. f. asiae genetic references (Fig 4). Subspecies assignment: QI, F. f. bogdanovi; QII, F. f. asiae; QIII, F. f. henrici (Fig 4: green, red and blue, respectively). (PDF) [file pone.0205059.s006.pdf]

**S6 Table**. **Posterior probability values from Bayesian analysis**. Posterior probability of membership to each of the three clusters as inferred by structure for (i) all single populations (with number, see Fig 1 and S1 Table), (ii) North, Central and South Pakistan, and (iii) *F. f. bogdanovi* and *F. f. asiae* genetic references (Fig 4). Subspecies assignment: $Q_{I}$, *F. f. bogdanovi*; $Q_{II}$, *F. f. asiae*; $Q_{III}$, *F. f. henrici* (Fig 4: green, red and blue, respectively).

| **Number** | **Population** | **All** | | | **Males** | | | **Females** | | |
|---|---|---|---|---|---|---|---|---|---|---|
| | | $Q_{I}$ | $Q_{II}$ | $Q_{III}$ | $Q_{I}$ | $Q_{II}$ | $Q_{III}$ | $Q_{I}$ | $Q_{II}$ | $Q_{III}$ |
| 1 | Jiwani | 0.473 | 0.420 | 0.107 | 0.803 | 0.129 | 0.067 | - | - | - |
| 2 | Karachi | 0.268 | 0.100 | 0.633 | - | - | - | 0.467 | 0.081 | 0.452 |
| 3 | Badeen | 0.302 | 0.173 | 0.525 | 0.196 | 0.095 | 0.708 | 0.252 | 0.259 | 0.489 |
| 4 | Chagai | 0.761 | 0.098 | 0.141 | 0.604 | 0.058 | 0.338 | 0.103 | 0.021 | 0.876 |
| 5 | Larkana | 0.196 | 0.093 | 0.711 | 0.427 | 0.072 | 0.502 | - | - | - |
| 6 | Jacobabad | 0.447 | 0.226 | 0.326 | 0.287 | 0.274 | 0.439 | 0.042 | 0.035 | 0.923 |
| 7 | Ghotki | 0.223 | 0.250 | 0.527 | 0.063 | 0.417 | 0.520 | 0.859 | 0.032 | 0.109 |
| 8 | Quetta | 0.884 | 0.028 | 0.087 | 0.848 | 0.027 | 0.125 | 0.082 | 0.012 | 0.906 |
| 9 | Zhoab | 0.686 | 0.040 | 0.274 | 0.455 | 0.042 | 0.503 | - | - | - |
| 10 | Haroon Abad | 0.818 | 0.047 | 0.135 | 0.264 | 0.085 | 0.651 | - | - | - |
| 11 | Basti | 0.597 | 0.239 | 0.164 | - | - | - | 0.312 | 0.186 | 0.502 |
| 12 | Alipur | 0.423 | 0.086 | 0.491 | 0.218 | 0.049 | 0.733 | 0.967 | 0.018 | 0.015 |
| 13 | Rakhni | 0.923 | 0.030 | 0.047 | 0.242 | 0.129 | 0.629 | 0.621 | 0.025 | 0.354 |
| 14 | Mekhtar | 0.729 | 0.053 | 0.219 | 0.775 | 0.065 | 0.161 | 0.021 | 0.025 | 0.954 |
| 15 | Musa | 0.857 | 0.054 | 0.089 | 0.508 | 0.137 | 0.356 | 0.914 | 0.007 | 0.079 |
| 16 | Ghazi Ghat | 0.083 | 0.056 | 0.861 | 0.090 | 0.049 | 0.861 | - | - | - |
| 17 | Bait Suvai | 0.372 | 0.062 | 0.566 | 0.521 | 0.040 | 0.439 | 0.471 | 0.036 | 0.493 |
| 18 | Chakwal | 0.429 | 0.032 | 0.539 | 0.674 | 0.023 | 0.304 | 0.232 | 0.017 | 0.752 |
| 19 | Sialkot | 0.222 | 0.090 | 0.688 | 0.627 | 0.046 | 0.328 | - | - | - |
| 20 | Bhimbar | 0.095 | 0.794 | 0.110 | - | - | - | 0.019 | 0.486 | 0.495 |
| 21 | Mirpur | 0.022 | 0.933 | 0.045 | - | - | - | 0.013 | 0.934 | 0.053 |
| 22 | Kotli | 0.016 | 0.940 | 0.044 | - | - | - | 0.014 | 0.934 | 0.052 |
| 23 | Kohala | 0.239 | 0.298 | 0.463 | 0.396 | 0.124 | 0.479 | - | - | - |
| 24 | Tandali | 0.021 | 0.955 | 0.024 | - | - | - | 0.017 | 0.955 | 0.028 |
| 25 | Muzaffarabad | 0.160 | 0.015 | 0.825 | 0.517 | 0.014 | 0.469 | - | - | - |
| - | North Pakistan | 0.150 | 0.507 | 0.342 | 0.553 | 0.051 | 0.395 | 0.059 | 0.665 | 0.276 |
| - | Central Pakistan | 0.571 | 0.097 | 0.331 | 0.408 | 0.111 | 0.481 | 0.439 | 0.039 | 0.521 |
| - | South Pakistan | 0.348 | 0.231 | 0.422 | 0.499 | 0.112 | 0.388 | 0.359 | 0.169 | 0.471 |
| - | *F. f. bogdanovi* | 0.896 | 0.011 | 0.093 | 0.895 | 0.027 | 0.078 | 0.955 | 0.011 | 0.034 |
| - | *F. f. asiae* | 0.039 | 0.926 | 0.035 | 0.020 | 0.914 | 0.066 | 0.042 | 0.893 | 0.066 |
